# High risk pregnancy associated perinatal morbidity and mortality: a second birth population-based survey in Huai’an in 2015

**DOI:** 10.1186/s12884-019-2323-6

**Published:** 2019-07-03

**Authors:** Xiaoqin Zhu, Huiyuan Niu, Hui Wang, Xiaoqiong Li, Tingting Qi, Weijie Ding, Liangrong Han, Muling Zhang, Honghua Guan, Shouzhong Li, Chunhong Tang, Yaodong Yin, Xihui Cao, Hong Liu, Cui Gao, Hongni Yue, Bo Sun

**Affiliations:** 1 Unit of Population Health Information, Departments of Obstetrics and Neonatology, Huai’an Women and Children’s Hospital, Huai’an, 223002 Jiangsu China; 2 Departments of Obstetrics and Pediatrics, Huai’an First General Hospital, Huai’an, 223002 Jiangsu China; 3 Departments of Obstetrics and Pediatrics, Huai’an Second General Hospital, Huai’an, 223002 Jiangsu China; 4 Departments of Obstetrics and Pediatrics, Huaiyin District Hospital, Huai’an, 223300 Jiangsu China; 5 Departments of Obstetrics and Pediatrics, Chuzhou District Hospital, Huai’an, 223200 Jiangsu China; 6 Departments of Obstetrics and Pediatrics, Lianshui County Hospital, Huai’an, 223400 Jiangsu China; 7 Departments of Obstetrics and Pediatrics, Xuyi County Hospital, Huai’an, 211700 Jiangsu China; 8 Departments of Obstetrics and Pediatrics, Hongze County Hospital, Huai’an, 223100 Jiangsu China; 9 Departments of Obstetrics and Pediatrics, Jinhu County Hospital, Huai’an, 211600 Jiangsu China; 10 Departments of Pediatrics and Neonatology, Children’s Hospital of Fudan University, 399 Wan Yuan Road, Shanghai, 201102 China

**Keywords:** High risk, Morbidity, Mortality, Perinatology, Neonate, Outcome, Pregnancy

## Abstract

**Background:**

The objective of this survey was to explore the association between pregnancy complications and perinatal outcome from regionally total birth population.

**Methods:**

In this prospectively collected data of complete birth registries from all level I-III hospitals in Huai’an in 2015, perinatal morbidity and mortality in relation to pregnancy complications and perinatal outcome were analyzed using international definitions. The results were compared with that of 2010 survey in the same region.

**Results:**

Of 59,424 total births in the hospitals of level I (*n* = 85), II (16) and III (6), delivery rate was 30.4, 40.1 and 29.5%, and rates of pregnancy complications were 12.9, 9.8 and 21.1% (average 14.1%), with antenatal corticosteroids rate in < 37 gestational weeks being 17.3, 31.0 and 39.9% (mean 36.6%), respectively. The preterm birth rate was 0.6, 2.7 and 9.5% (mean 4.06%), and the composite rate of fetal death, stillbirth, and death immediately after delivery was 0.1, 0.4 and 0.6%, respectively. By multivariable logistic regression analysis, congenital anomalies, low Apgar scores, multi-pregnancy and amniotic fluid contamination were risk factors of adverse perinatal outcomes. Despite a higher rate of pregnancy complications than in 2010 survey, perinatal and neonatal mortality continued to fall, in particular in very preterm births. The high cesarean delivery rate in non-medically indicated cases remained a challenge.

**Conclusions:**

Our regional birth-population data in 2015 revealed a robust and persistent improvement in the perinatal care and management of high risk pregnancies and deliveries, which should enable more studies using similar concept and protocol for vital statistics to verify the reliability and feasibility.

**Electronic supplementary material:**

The online version of this article (10.1186/s12884-019-2323-6) contains supplementary material, which is available to authorized users.

## Background

The outcome of neonates undergoing hospitalization at early postnatal life is associated with prenatal and postnatal morbidities [1–4]. With medical advances in both obstetric care for high-risk pregnancies and neonatal intensive care, the survival rate of preterm and critically ill neonates has been steadily increased in many regions of China [1, 5–10]. This progress has been facilitated by the implementation of a nationwide universal health insurance, namely “New Rural Cooperative Medical Scheme” for hospital deliveries and hospitalized neonates since 2010 [2, 11]. However, the relationship between pregnancy, or perinatally-associated, co-morbidities and complications (shortly referred as pregnancy complications in below text) and the outcome of hospitalized neonates remain to be clarified in sub-provincial regions. These regions constitute a large part of China and are responsible for the majority deliveries nationwide, with otherwise relatively underdeveloped perinatal-neonatal care infrastructures compared to economically advantageous coastal regions surrounding prosperous metropolitan cities. Our 2010 Huai’an survey was hitherto the very first reporting on co-morbidities and complications in all pregnancies, perinatal and neonatal mortality, preterm birth rate and preterm infants mortality in the sub-provincial birth-population level [12, 13]. Given the incompleteness and inaccuracy of current official vital statistics on all births not including those neonates with a gestational age < 28 weeks [6, 14], our concept and methodology of regional birth-population based registry may provide a valuable tool for assessing quality of maternal-fetal care and perinatal and neonatal outcomes. In this regard, we conducted a second phase complete birth population-based survey in 2015 [15], and evaluated the association between pregnancy complications and perinatal and neonatal death rates [15]. We hypothesized that with further improvement in maternal health care facilitated by universal health insurance, more pregnancy complications might be timely identified and managed at level II and III facilities, and contributed to a continued reduction of perinatal and neonatal mortality [12, 13]. Our purpose of the current study was to comprehensively explore the impact of pregnancy complications on perinatal and neonatal mortality, categorized by hospital levels, delivery modes as well as maternal age and education years as socioeconomic status. A thorough understanding in these aspects will substantially facilitate advances in the overall perinatal-neonatal care and quality improvement of the region.

## Methods

### Study region and population

The survey was conducted in Huai’an city, with a population of 5,400,000-5,600,000 and around 60,000 annual births in 2009–2014 [16]. Approximately 5% of the population was migrants from other regions. Of the total population, 55% were urban residents and the remaining lived in the rural area. The regional economic status, evaluated by gross domestic production per capita (GDP), is similar to the national average level in 2015 [16]. The socio-cultural background in this region does not differ from that in most east and mid-land provinces of China. The rate of hospital delivery in this region is > 99%, and since 2010 almost all rural residents and their newborns have been covered by the universal health insurance [12, 15, 17].

### Study design, protocol and ethical approval

Study setting: 107 level I-III hospitals (Table 1) providing obstetric care, with 8 in level II and 4 in level III equipped with neonatal wards (For the name list of level II and III hospitals as the member of Huai’an Perinatal-Neonatal Study Group see Additional file 1).

**Table 1:** Comparison of all births, pregnancy co-morbidity and complications, and fetal and neonatal deaths in different levels of hospitals in Huai’an in 2015

Hospital level	I	II	III	Total
Number of hospitals	85	16	6	
All births	17,991 (30.4)	23,758 (40.1)	17,441 (29.5)	59,190
Fetal deaths/stillbirths	9 (0.1)	78 (0.3)	80 (0.5)	167 (0.28)
Live births	17,982 (99.9)	23,680 (99.7)	17,361 (99.5)	59,023 (99.7)
Males*	9959 (55.4)	12,278 (51.7)	9259 (53.1)	31,496 (53.2)
Gestational age (weeks)*	39.3 ± 1.1	39.2 ± 1.3	38.7 ± 2.0	39.1 ± 1.5
Birthweight (grams)*	3480 ± 456	3465 ± 476	3392 ± 587	3448 ± 507
Preterm births*	111 (0.6)	639 (2.7)	1654 (9.5)	2404 (4.06)
Low birthweight*	179 (1.0)	437 (1.8)	1079 (6.2)	1695 (2.9)
Multiple-births*	101 (0.6)	426 (1.8)	723 (4.1)	1250 (2.1)
Congenital anomalies^a^*	80 (0.4)	165 (0.7)	79 (0.5)	324 (0.55)
Cesarean delivery*	9265 (51.5)	13,778 (58.0)	9183 (52.7)	32,226 (54.4)
Pregnancy complications*	2322 (12.9)	2331 (9.8)	3675 (21.1)	8328 (14.1)
HDP*	541 (3.0)	1155 (4.9)	1224 (7.0)	2920 (4.9)
PROM*	773 (4.3)	2303 (9.7)	2191 (12.6)	5267 (8.9)
GDM	62 (0.3)	112 (0.5)	730 (4.2)	904 (1.5)
Anemia*	1116 (6.2)	1096 (4.6)	2272 (13.0)	4484 (7.6)
Antenatal steroids*^f^	17 (17.3)	189 (31.0)	638 (39.9)	844 (36.6)
Maternal age*, years	26.6 ± 5.0	26.8 ± 4.7	27.4 ± 4.8	26.9 ± 4.8
Delayed childbearing*	1428 (7.9)	1842 (7.8)	1646 (9.4)	4916 (8.3)
> 9 years’ education*	1967 (10.9)	8926 (37.6)	11,846 (67.9)	22,739 (38.4)
Amniotic fluid volume*				
Normal	15,125 (84.1)	20,697 (87.1)	14,539 (83.4)	50,361 (85.1)
Polyhydramnios	1603 (8.9)	865 (3.6)	1720 (9.9)	4188 (7.1)
Oligohydramnios	1263 (7.0)	2196 (9.2)	1182 (6.8)	4641 (7.8)
Amniotic contamination*				
Normal	15,847 (88.1)	19,808 (83.4)	15,416 (88.4)	51,071 (86.3)
Grade I	1080 (6.0)	1464 (6.2)	380 (2.2)	2924 (4.9)
Grade II	623 (3.5)	1214 (5.1)	385 (2.2)	2222 (3.8)
Grade III	441 (2.5)	1272 (5.4)	1260 (7.2)	2973 (5.0)
Apgar score				
1-min ≤7*	181 (1.0)	533 (2.2)	857 (4.9)	1571 (2.7)
1-min ≤3*	36 (0.2)	156 (0.7)	155 (0.9)	347 (0.6)
5-min ≤7*	68 (0.4)	199 (0.8)	287 (1.6)	554 (0.9)
5-min ≤3*	44 (0.2)	126 (0.5)	123 (0.7)	293 (0.5)
Deaths at delivery^b^*	12 (0.1)	88 (0.4)	98 (0.6)	198 (0.3)
Hospitalized on day 1*	0	1162 (4.9)	2103 (12.1)	3265 (5.7)
Early neonatal deaths^c^*	21 (0.1)	40 (0.2)	78 (0.4)	139 (0.2)
Late neonatal deaths^d^*	6 (0.03)	10 (0.04)	25 (0.1)	41 (0.1)
All deaths^e^*	37 (0.2)	131 (0.6)	190 (1.1)	358 (0.6)
Perinatal mortality*, n (‰)	30 (1.6)	118 (5.0)	158 (9.1)	306 (5.2)
Neonatal mortality*, n (‰)	27 (1.5)	50 (2.1)	103 (5.9)	180 (3.0)

All values are given in numbers and percentage (% of all births in each hospital level category) or means ± SD

*Abbreviations*: *HDP* Hypertensive disorders of pregnancy, including hypertension, eclampsia and preeclampsia, *PROM* Premature rupture of membrane, including 12.9% as preterm births, *GDM* Gestational diabetes mellitus, including pre-pregnancy DM

*P < 0.01 for between-group differences

^a^Including all birth defects and congenital disorders from total births within 7 postnatal days

^b^Including fetal deaths/stillbirth and neonatal deaths immediately at delivery but counted as live births

^c^Including live births died in the first 7 days

^d^Including live births died in the 8 to 28 days after delivery

^e^Including fetal deaths/stillbirth and live births deaths after birth. Perinatal mortality includes all fetal deaths/stillbirth from 22 complete gestational week and neonatal deaths in the first 7 postnatal days in reference to total births. Neonatal mortality includes all deaths of live birth in 0–28 postnatal days in reference to the total live births

^f^Data contained cases referenced to total preterm births (*n* = 2404)

Study design: prospective, cross-sectional survey of complete birth data from hospital deliveries with information on pregnancy complications and perinatal morbidities and mortalities.

Study population: all births (from gestational age of 22 complete weeks) [18, 19] including fetal death, stillbirth and live births collected consecutively from Jan 1 to Dec 31, 2015. Non-medical indication related end-of-pregnancy was not included.

Sample size: a sample size of 60,000 was estimated based on regional annual birth data in 2009–2014, including deliveries from migrants without registered residency.

Data collection: for all hospital deliveries, clinical data were extracted from case records through the regional perinatal-neonatal information system by co-investigators and task force group members from all level I-III hospitals in the regional hospital network [12, 15].

Study variables: maternal characteristics included age, education years and socioeconomic status, as well as pregnancy complications, and perinatal and neonatal morbidity and mortality. Neonatal biological and pathological characteristics included sex, gestational age (GA), birthweight (BW), perinatal co-morbidities and death.

The study protocol was approved by the Ethics Committee of Huai’an Women and Children’s Hospital. As data were collected from observational parameters and no specific intervention was used, the Ethics Committee approval waived informed consent. All the participating level II and III hospitals were invited as members of the Huai’an Perinatal-Neonatal Study Group (name list see Additional file 1: Table S1), and each hospital administrative body, as well as the departments, adopted and approved the Ethics Approval and the study protocol through official agreement, and complied by the domestic regulations for clinical practice and investigation (www.nch.gov.cn).

### Definitions of vital statistics and perinatal morbidities

Live birth, fetal death, stillbirth, or death during delivery are defined according to the 10th revision of the international classification of diseases [18]. GA, BW and perinatal and neonatal death rate are described elsewhere [12, 13, 15]. Low birth weight is defined as birth weight < 2500 g, and preterm as GA < 37 complete weeks (< 259 days) [19]. To enable comparison with vital statistics from middle income or upper middle income countries by the World Bank classification, the perinatal period was defined as 22 complete weeks (154 days) of GA to 7 days postnatally and the neonatal period is the first 28 complete days after birth. As consistent with previous studies [12, 13, 15], fetal death is synonymous with stillbirth, and is defined as born deaths (including fetal death and death during delivery but not immediately after birth, or Apgar score 0) per 1000 total births. Perinatal mortality refers to the deaths in perinatal period per 1000 total births. Neonatal mortality refers to the neonatal deaths (including deaths immediately after birth in the delivery room), in the first 28 days of postnatal life, per 1000 live births (again, including those died immediately after birth but with life signs at birth, or Apgar score ≥ 1). Death immediately after birth was defined for those requiring resuscitation but died within few minutes in the delivery room, often at the request of parents to discontinue resuscitation or withhold/withdraw rescue intervention. The diagnosis criteria of pregnancy complications were based on international definitions [20]. Perinatal morbidities, birth defects (BD) or congenital anomalies (CA) identified within the first 7 postnatal days, and major neonatal diseases were defined according to Fanaroff and Martin [21] and domestic clinical criteria. Data presented as rates were calculated using the number of total births, total live births or pregnancies as the denominator where appropriate. Quality control was conducted by on-site physician/investigators with training and educational sessions to ensure the accuracy and completeness of data retrieval [15]. Site visiting, telephone or e-mail communications were routinely carried out for verification and correction of the data. General periodical quality control inspections involving birth registration or medical records from all the level II and III (municipal and county) hospitals and 20% township hospitals were performed. Data of neonates transported to other tertiary centers (mainly in Nanjing, the capital of the province) were also retrieved. Major findings were compared with those of the 2010 survey [12, 13].

### Statistical analysis

The EPIDATA database was used for datasheet recordings and statistical analysis was performed using SPSS software (v. 16.0, SPSS Inc. Chicago, IL). Continuous variables were presented by the mean and standard deviation (SD), or median and interquartile range (IQR) where appropriate, being analyzed by one-way analysis of variance for comparisons between-group differences. Categorical variables were presented as frequencies or rates, using Chi square test for comparison of differences. A *p* value < 0.05 was considered statistically significant. For assessment of relative risks of perinatal mortality, uni- and multivariable logistic regression analysis was performed for binary or categorical data. Values were presented as odds ratio (OR) with 95% confidence interval (CI).

## Results

### Vital statistics

There were a total of 59,424 birth registries and 59,190 had complete perinatal information. Of them, 59,023 (99.7%) were live births and 167 were stillbirths (fetal deaths). The total birth rate was 10.5‰ (the regional population was 5644,500 in 2015). Table 1 illustrates maternal-fetal and neonatal baseline characteristics in three levels of the hospitals providing deliveries. Nearly 70% of deliveries took place in level II and III hospitals, which increased significantly compared to the 2010 survey (47.7%, χ^2^ = 62.9, *p* < 0.001) [13]. Preterm infants with low BW, pregnancy complications, BD/CA, fetal death/stillbirth and perinatal and neonatal mortality were overrepresented in level II and III hospitals, demonstrating highly centralized management of high risk pregnancies and deliveries. There were 31 (0.5‰) neonatal deaths at delivery, compared to 106 (1.7‰) in 2010 survey. Similarly, the rate of fetal death/stillbirth demonstrated 1.1‰ (2.8 vs. 3.9‰) or a nearly 30% relative decline as compared to 2010 [15]. Compared to the data in 2010 [12, 13], the rates of BD/CA, Apgar score ≤ 3 at 1 and 5 min, and neonatal death were moderately declined, but more infants were born with abnormal amniotic fluid in 2015. Additionally, fewer mothers with delayed age of child bearing (≥ 35 years old) were observed in 2015, accompanied by more mothers with education > 9 years. Compared to 2010 survey, an increased number of preterm infants were born in level III hospitals, both in the absolute number and the delivery rate in preterm population [(1059/2239, 47.3%) in 2010 vs. (1654/2404, 68.8%) in 2015, χ^2^ = 220.7, *p* < 0.001]. A trend of increasing use of antenatal corticosteroids in preterm infants with GA < 37 weeks was found in all three level hospitals.

The perinatal and neonatal mortality was 5.2‰ (167 stillbirths/fetal death, 138 early neonatal deaths including 31 deaths at delivery) and 3.0‰ (180), respectively [15]. The main causes of perinatal mortality for 211 stillbirths and early neonatal deaths with ante- and intrapartum risks were BD/CA (42, 19.9%), intrauterine distress (31, 14.7%), birth asphyxia (18, 8.5%), hypertensive disorders of pregnancy (HDP) and other complications (11, 5.2%), abnormal umbilical cord (nuchal cord, funisitis) (10, 4.7%) and placenta (placenta previa, abruption) (7, 3.3%) and sepsis (4, 1.9%). In the neonatal deaths 87 (48.2%) were preterm, and 57 (31.7%) were very preterm (< 33 weeks of gestation). Of the 136 hospitalized neonatal deaths caused by parents’ decisions of treatment withhold/withdraw, 37.9% were related to deterioration of underlying diseases, 31.8% to worse prognosis for long-term outcomes, 16.7% resulted from severe malformations, 12.1% due to economic reasons, and 1.5% for other reasons.

### Pregnancy complications

Pregnancy complications, such as premature rupture of membrane (5267, 8.9%), HDP (2920, 4.9%), anemia (4522, 7.6%), preeclampsia (2525, 4.3%), placenta previa (388, 0.6%), hepatitis (2355, 4.0%) and diabetes (904, 1.5%) were recorded in 14.1% of total births. Table 2 compares the maternal and perinatal characteristics between births with recorded pregnancy complications and those without. The rate of cesarean delivery, fetal death/stillbirth, preterm birth, low BW, multiple births, BD/CA, death at delivery, and neonatal death were significantly higher in those with pregnancy complications than those without.

**Table 2:** Pregnancy co-morbidities and complications related maternal, fetal and neonatal status of all births

	Pregnancy co-morbidities and complications		
	Yes	No	Total
All pregnancies	8188 (14.0)	50,374 (86.0)	58,562
Maternal age (years)*	27.2 ± 5.0	26.9 ± 4.8	26.9 ± 4.9
< 20	275 (3.3)	1695 (3.3)	1970 (3.3)
20–24	2181 (26.2)	14,568 (28.6)	16,749 (28.3)
25–29	3659 (43.9)	22,613 (44.5)	26,272 (44.4)
30–34	1391 (16.7)	7892 (15.5)	9283 (15.7)
≥ 35	822 (9.9)	4094 (8.0)	4916 (8.3)
Antenatal steroids^f^	203 (33.7)	641 (37.7)	844 (1.4)
First delivery*	5835 (70.1)	34,065 (67.0)	39,900 (67.4)
Cesarean delivery*	4786 (57.5)	27,440 (53.9)	32,226 (54.4)
Birth numbers	8328 (14.1)	50,862 (85.9)	59,190
Live births	8294 (99.6)	50,729 (99.7)	59,023 (99.7)
Preterm births*	621 (7.5)	1783 (3.5)	2404 (4.06)
24–27 weeks	12 (0.1)	36 (0.1)	48 (0.08)
28–31 weeks	68 (0.8)	200 (0.4)	268 (0.45)
32–36 weeks	541 (6.5)	1547 (3.0)	2088 (3.53)
Full term births*	7625 (91.6)	48,534 (95.4)	56,159 (94.9)
37–38 weeks	1980 (23.8)	11,128 (21.9)	13,108 (22.1)
39–41 weeks	5645 (67.8)	37,406 (73.5)	43,051 (72.7)
Post-term births	82 (1.0)	545 (1.1)	627 (1.06)
LBW	391 (4.7)	1304 (2.6)	1695 (2.86)
VLBW*	52 (0.6)	171 (0.3)	223 (0.4)
ELBW	8 (0.1)	30 (0.1)	38 (0.1)
Multi-births*	276 (3.3)	974 (1.9)	1250 (2.11)
Congenital anomalies^a^	49 (0.6)	275 (0.5)	324 (0.5)
Amniotic fluid volume*			
Normal	7271 (87.3)	43,090 (84.7)	50,361 (85.1)
Polyhydramnios	408 (4.9)	3780 (7.4)	4188 (7.1)
Oligohydramnios	649 (7.8)	3993 (7.9)	4642 (7.8)
Amniotic contamination*			
Normal	7122 (85.5)	43,949 (86.4)	51,071 (86.3)
Grade I	388 (4.7)	2536 (5.0)	2924 (4.9)
GradeII	270 (3.2)	1952 (3.8)	2222 (3.8)
Grade III	548 (6.6)	2425 (4.8)	2973 (5.0)
Apgar score			
1-min ≤7*	375 (4.5)	1196 (2.4)	1571 (2.7)
1-min ≤3*	70 (0.8)	277 (0.5)	347 (0.6)
5-min ≤7*	113 (1.4)	441 (0.9)	554 (0.9)
5-min ≤3*	55 (0.7)	238 (0.5)	293 (0.5)
Fetal deaths/stillbirths*	34 (0.4)	133 (0.3)	167 (0.3)
Deaths at delivery^b^*	42 (0.5)	156 (0.3)	198 (0.3)
Hospitalized on day 1*	675 (8.1)	2589 (5.1)	3264 (5.5)
Early neonatal deaths^c^	26 (0.3)	113 (0.2)	139 (0.2)
Late neonatal deaths^d^	5 (0.06)	36 (0.07)	41 (0.07)
All deaths^e^*	66 (0.8)	292 (0.6)	358 (0.6)

All values are given in numbers and percentage (% of all pregnancies or birth numbers) or means ± SD

*Abbreviations*: *LBW* Low birth weight (< 2500 g), *VLBW* Very LBW (< 1500 g), *ELBW* Extremely LBW (< 1000 g)

*P < 0.01 for between-group differences

^a^Including all birth defects and congenital disorders from total births within 7 postnatal days

^b^Including fetal deaths/stillbirth and neonatal deaths immediately at delivery but counted as live births

^c^Including live births died in the first 7 days

^d^Including live births died in the 8 to 28 days after delivery

^e^Including all fetal deaths/stillbirth and all neonatal deaths

^f^Data contain all premature babies (< 37 gestational weeks) received antenatal corticosteroid therapy before delivery

Tables 3, 4 and 5 illustrate pregnancy complications categorized by BW, GA and delivery mode, respectively. Tables 6 and 7 show perinatal morbidities and mortalities categorized by maternal age or education years, respectively. It is obvious that mothers with delayed child bearing (i.e. age ≥ 35 years) were more likely than younger (< 20 years) mothers to have preterm and low birth weight infants. This survey also indicated that < 1% of all mothers were illiterate, mothers with education > 9 years were more likely to develop pregnancy complications, be admitted to level III hospitals, have severely contaminated amniotic fluid, and deliver preterm and/or low birth weight infants than mothers who were less educated. However, cesarean section, low Apgar score, neonatal deaths (including death at delivery) and BD/CA did not seem to be more prevalent in the mothers with education > 9 years than those who were less educated.

**Table 3:** Perinatal status and pregnancy co-morbidities and complications in birthweight strata of all births

	Birthweight (g)				
	< 1500	1500-2499	2500-3999	≥4000	Total
Birth number (%)	223 (0.4)	1472 (2.5)	49,268 (83.2)	8227 (13.9)	59,190
Males	124 (55.6)	718 (48.8)	25,509 (51.8)	5145 (62.5)	31,496 (53.2)
Gestational age (weeks)*	30.3 ± 3.8	35.3 ± 2.8	39.1 ± 1.2	39.6 ± 1.0	39.1 ± 1.5
Birthweight (grams)	1164 ± 217	2114 ± 267	3370 ± 333	4220 ± 281	3448 ± 507
Preterm birth*	199 (89.2)	951 (64.6)	1231 (2.5)	23 (0.3)	2404 (4.1)
Multi-births*	66 (29.6)	411 (27.9)	770 (1.6)	3 (0.04)	1250 (2.1)
Congenital anomalies^a^*	6 (2.7)	43 (2.9)	243 (0.5)	32 (0.4)	324 (0.5)
Cesarean delivery*	100 (44.8)	891 (60.5)	25,790 (52.3)	5445 (66.2)	32,226 (54.4)
Pregnancy complications*	52 (23.3)	339 (23.0)	6716 (13.6)	1221 (14.8)	8328 (14.1)
HDP*	40 (17.9)	247 (16.8)	2138 (4.3)	495 (6.0)	2920 (4.9)
PROM*	52 (23.3)	290 (19.7)	4262 (8.7)	663 (8.1)	5267 (8.9)
GDM*	4 (1.8)	38 (2.6)	650 (1.3)	212 (2.6)	904 (1.5)
Anemia*	32 (14.3)	212 (14.4)	3540 (7.2)	700 (8.5)	4484 (7.6)
Antenatal steroids^f^	101 (57.7)	448(48.5)	292(24.6)	3(13.6)	844 (36.6)
Maternal age (years)	27.3 ± 5.4	27.0 ± 5.3	26.8 ± 4.8	27.7 ± 5.0	26.9 ± 4.8
Delayed childbearing*	26 (11.7)	147 (10.0)	3841 (7.8)	902 (11.0)	4916 (8.3)
> 9 years’ education*	120 (53.8)	658 (44.7)	18,892 (38.3)	3069 (37.3)	22,739 (38.4)
Amniotic fluid volume*					
Normal	193 (86.5)	1263 (85.8)	41,870 (85.0)	7035 (85.5)	50,361 (85.1)
Polyhydramnios	1 (0.4)	28 (1.9)	3443 (7.0)	716 (8.7)	4188 (7.1)
Oligohydramnios	30 (13.5)	181 (12.3)	3955 (8.0)	476 (5.8)	4642 (7.8)
Amniotic contamination*					
Normal	192 (86.1)	1326 (90.1)	42,572 (86.4)	6981 (84.9)	51,071 (86.3)
Grade I	8 (3.6)	50 (3.4)	2413 (4.9)	453 (5.5)	2924 (4.9)
GradeII	6 (3.0)	25 (1.7)	1828 (3.7)	363 (4.4)	2222 (3.8)
Grade III	17 (7.6)	71 (4.8)	2455 (5.0)	430 (5.2)	2973 (5.0)
Apgar score*					
1-min ≤7	167 (74.9)	365 (24.8)	914 (1.9)	125 (1.5)	1571 (2.7)
1-min ≤3	69 (30.9)	97 (6.6)	160 (0.3)	21 (0.3)	347 (0.6)
5-min ≤7	112 (50.2)	141 (9.6)	259 (0.5)	42 (0.5)	554 (0.9)
5-min ≤3	52 (23.3)	71 (4.8)	139 (0.3)	31 (0.4)	293 (0.5)
Hospitalized on day 1*	154 (69.1)	864 (58.7)	1971 (4.0)	275 (3.3)	3264 (5.5)
IVH/PVH*	70 (40.5)	418 (44.9)	388 (19.2)	35 (12.6)	911 (26.8)
RDS*	104 (61.1)	106 (11.4)	60 (3.0)	2 (0.7)	272 (8.0)
Ventilation*	119 (68.8)	263 (28.3)	245 (12.1)	26 (9.4)	653 (19.2)
Fetal deaths/stillbirths*	35 (15.7)	61 (4.1)	61 (0.1)	10 (0.1)	167 (0.3)
Deaths at birth^b^	52 (23.3)	66 (4.5)	70 (0.1)	10 (0.1)	198 (0.3)
Early neonatal deaths^c^*	36 (16.1)	26 (1.8)	71 (0.1)	6 (0.1)	139 (0.2)
Late neonatal deaths^d^	6 (2.7)	9 (0.6)	20 (0.04)	6 (0.1)	41 (0.1)
All deaths^e^	82 (36.8)	97 (6.6)	157 (0.3)	22 (0.3)	358 (0.6)

All values are numbers and percentage (% of total births in each birthweight category) or means ± SD

*Abbreviations*: *HDP* Hypertensive disorders of pregnancy, *PROM* Premature rupture of membrane, *GDM* Gestational diabetes mellitus, *IVH/PVH* Intraventricular/perinventricular hemorrhage, *RDS* Respiratory distress syndrome

*p < 0.001

^a^Including all birth defects and congenital disorders from total births within 7 postnatal days

^b^Including fetal deaths/stillbirth and neonatal deaths immediately at delivery but counted as live births

^c^Including live births died in the first 7 days

^d^Including live births died in the 8 to 28 days after delivery

^e^Including all fetal deaths/stillbirth and all neonatal deaths

^f^Data contain all premature babies received antenatal corticosteroid therapy before delivery

**Table 4:** Perinatal status and pregnancy co-morbidities and complications in gestational age strata of all births

Gestational age (weeks)	< 32	32–36	37–41	≥42	Total
All births	316 (0.5)	2088 (3.5)	56,159 (94.9)	627 (1.1)	59,190
Males*	191 (60.4)	1186 (56.8)	29,817 (53.1)	302 (48.2)	31,496 (53.2)
Gestational age (weeks)	29.2 ± 1.6	34.9 ± 1.3	39.2 ± 1.0	42.1 ± 0.3	39.1 ± 1.5
Birthweight (g)	1464 ± 443	2583 ± 546	3489 ± 451	3677 ± 483	3448 ± 507
LBW*	310 (98.1)	840 (40.2)	544 (1.0)	1 (0.2)	1695 (2.9)
Multi-births*	79 (25.0)	482 (23.1)	689 (1.2)	0 (0.0)	1250 (2.1)
Congenital anomalies^a^*	16 (5.1)	38 (1.8)	267 (0.5)	3 (0.5)	325 (0.5)
Cesarean delivery*	115 (36.4)	1296 (62.1)	30,463 (54.2)	352 (56.1)	32,226 (54.4)
Pregnancy complications*	80 (25.3)	541 (25.9)	7625 (13.6)	82 (13.1)	8328 (14.1)
HDP*	39 (1.3)	307 (10.5)	2535 (86.8)	39 (1.3)	2920 (4.9)
PROM*	81 (25.6)	598 (28.6)	4554 (8.1)	34 (5.4)	5267 (8.9)
GDM*	9 (2.8)	96 (4.6)	796 (1.4)	3 (0.5)	904 (1.5)
Anemia	51 (16.1)	347 (16.6)	4033 (7.2)	53 (8.5)	4484 (7.6)
Antenatal steroids^f^	164 (58.2)	680(33.6)	0	0	844 (36.6)
Maternal age (years)	27.4 ± 5.4	27.6 ± 5.4	26.9 ± 4.8	26.1 ± 5.1	26.9 ± 4.8
Delayed childbearing*	40 (12.7)	264 (12.6)	4565 (8.1)	47 (7.5)	4916 (8.3)
> 9 years’ education*	160 (50.6)	1082 (51.8)	21,363 (38.0)	134 (21.4)	22,739 (38.4)
Amniotic fluid volume*					
Normal	261 (82.6)	1842 (88.2)	47,717 (85.0)	541 (86.3)	50,361 (85.1)
Polyhydramnios	8 (2.5)	40 (1.9)	4136 (7.4)	4 (0.6)	4188 (7.1)
Oligohydramnios	48 (15.2)	206 (9.9)	4306 (7.7)	82 (13.1)	4642 (7.8)
Amniotic contamination*					
Normal	275 (87.0)	1949 (93.3)	48,362 (86.1)	485 (77.4)	51,071 (86.3)
Grade I	13 (4.1)	44 (2.1)	2811 (5.0)	56 (8.9)	2924 (4.9)
Grade II	9 (2.8)	21 (1.0)	2151 (3.8)	41 (6.5)	2222 (3.8)
Grade III	19 (6.0)	74 (3.5)	2835 (5.0)	45 (7.2)	2973 (5.0)
Apgar score					
1-min ≤ 7*	227 (71.8)	443 (21.2)	892 (1.6)	9 (1.4)	1571 (2.7)
1-min ≤ 3*	97 (30.7)	91 (4.4)	158 (0.3)	1 (0.2)	347 (0.6)
5-min ≤ 7*	155 (49.1)	133 (6.4)	262 (0.5)	4 (0.6)	554 (0.9)
5-min ≤3*	77 (24.4)	65 (3.1)	149 (0.3)	2 (0.3)	293 (0.5)
Hospitalized on day 1*	219 (69.3)	1191 (57.0)	1841 (3.3)	13 (2.1)	3264 (5.5)
IVH/PVH*	125 (52.1)	534 (40.9)	248 (13.5)	4 (30.8)	911 (26.8)
RDS*	132 (55.0)	104 (8.0)	36 (2.0)	0	272 (8.0)
Ventilation*	167 (69.6)	282 (21.6)	204 (11.1)	0	653 (19.2)
Fetal deaths/stillbirths*	55 (17.4)	58 (2.8)	54 (0.1)	0 (0)	167 (0.3)
Deaths at delivery^b^*	75 (23.7)	62 (3.0)	61 (0.1)	0	198 (0.3)
Early neonatal deaths^c^*	44 (13.9)	32 (1.5)	62 (0.1)	1 (0.2)	139 (0.2)
Late neonatal deaths^d^*	6 (1.9)	5 (0.2)	30 (0.1)	0	41 (0.1)
All deaths^e^*	110 (34.8)	95 (4.5)	151 (0.3)	2 (0.3)	358 (0.6)

Values are given in numbers and percentage (% of all births in each gestational age category) or mean ± SD. Percentage of total numbers of all births includes fetal death/stillbirths and neonatal death at delivery (see Table 1)

*Abbreviations*: *LBW* Low birth weight (< 2500 g), *HDP* Hypertensive disorders of pregnancy, *PROM* Premature rupture of membrane, *GDM* Gestational diabetes mellitus, *IVH/PVH* Intraventricular/perinventricular hemorrhage, *RDS* Respiratory distress syndrome

*P < 0.001

^a^Including all birth defects and congenital disorders from total births within 7 postnatal days

^b^Including fetal deaths/stillbirth and neonatal deaths immediately at delivery but counted as live births

^c^Including live births died in the first 7 days

^d^Including live births died in the 8 to 28 days after delivery

^e^Including all fetal deaths/stillbirth and all neonatal deaths

^f^Data contain all premature babies received antenatal corticosteroid therapy before delivery

**Table 5:** Mode of delivery related perinatal status, pregnancy co-morbidities and complications, fetal deaths/stillbirths and neonatal mortalities in all births

Mode of delivery	Vaginal**	Cesarean	Total
All births	26,964 (45.6)	32,226 (54.4)	59,190
Live births, n (%)	26,827 (99.5)	32,196 (99.9)	59,023 (99.7)
Males*	13,990 (51.9)	17,506 (54.3)	31,496 (53.2)
Gestational age* (weeks)	39.2 ± 1.6	39.0 ± 1.5	39.1 ± 1.5
Birthweight* (grams)	3409 ± 477	3481 ± 528	3448 ± 507
Preterm birth*	993 (3.7)	1411 (4.4)	2404 (4.1)
LBW*	704 (2.6)	991 (3.1)	1695 (2.9)
Multi-births*	233 (0.9)	1017 (3.2)	1250 (2.1)
Congenital anomalies^a^	149 (0.6)	175 (0.5)	324 (0.55)
Maternal age* (years)	26.4 ± 4.7	27.4 ± 4.9	26.9 ± 4.8
Delayed childbearing	1851 (1.0)	3065 (9.5)	4916 (8.3)
> 9 years education*	10,818 (40.1)	11,921 (37.0)	22,739 (38.4)
Pregnancy complications*	3542 (13.1)	4786 (14.9)	8328(14.1)
HDP*	532 (2.0)	2388 (7.4)	2920 (4.9)
PROM*	2163 (8.0)	3104(9.6)	5267 (8.9)
GDM*	307 (1.1)	597 (1.9)	904 (1.5)
Anemia*	1577 (5.8)	2907 (9.0)	4484 (7.6)
Antenatal corticosteroids^f^	313 (33.6)	531 (38.6)	844 (36.6)
Amniotic fluid volume*			
Normal	24,109 (89.4)	26,252 (81.5)	50,361 (85.1)
Polyhydramnios	1670 (6.2)	2518 (7.8)	4188 (7.1)
Oligohydramnios	1186 (4.4)	3456 (10.7)	4642 (7.8)
Amniotic contamination*			
Normal	23,358 (86.6)	27,713 (86.0)	51,071 (86.3)
Grade I	1247 (4.6)	1677 (5.2)	2924 (4.9)
Grade II	937 (3.5)	1285 (4.0)	2222 (3.8)
Grade III	1422 (5.3)	1551 (4.8)	2973 (5.0)
Apgar score			
1-min ≤7	755 (2.8)	816 (2.5)	1571 (2.7)
1-min ≤3*	226 (0.8)	121 (0.4)	347 (0.59)
5-min ≤7*	316 (1.2)	238 (0.7)	554 (0.94)
5-min ≤3*	205 (0.8)	88 (0.3)	293 (0.50)
Hospitalized on day 1*	1338 (5.0)	1926 (6.0)	3264 (5.5)
IVH/PVH	352 (24.5)	559 (28.5)	911 (26.8)
RDS	114 (7.9)	158 (8.1)	272 (8.0)
Ventilation*	236 (16.4)	417 (21.3)	653 (19.2)
Fetal deaths/stillbirths*	137 (0.5)	30 (0.1)	167 (0.3)
Deaths at delivery^b^*	160 (0.6)	38 (0.1)	198 (0.33)
Early neonatal deaths^c^	73 (0.2)	66 (0.2)	139 (0.23)
Late neonatal deaths^d^	15 (0.1)	26 (0.1)	41 (0.07)
All deaths^e^*	221 (0.8)	137 (0.4)	358 (0.6)

Values are given in numbers and percentage (% of all births in each delivery category) or mean ± SD. * Percentage of total numbers of all births includes fetal death/stillbirths and neonatal death at delivery (see Table 1)

*Abbreviations*: *LBW* Low birth weight (< 2500 g), *HDP* Hypertensive disorders of pregnancy, *PROM* Premature rupture of membrane, *GDM* Gestational diabetes mellitus, *IVH/PVH* Intraventricular/perinventricular hemorrhage, *RDS* Respiratory distress syndrome

* *p* < 0.001; ** including 397 with assisted operation procedures

^a^Including all birth defects and congenital disorders from total births within 7 postnatal days

^b^Including fetal deaths/stillbirth and neonatal deaths immediately at delivery but counted as live births

^c^Including live births died in the first 7 days

^d^Including live births died in the 8 to 28 days after delivery

^e^Including all fetal deaths/stillbirth and all neonatal deaths

^f^Data contain all preterm babies received antenatal corticosteroid therapy before delivery

**Table 6:** Maternal age related perinatal status, pregnancy co-morbidities and complications, fetal deaths/stillbirths and neonatal mortality in all births

Maternal age, years old	< 20	20–34	≥35	Total
Maternal age (years)	18.2 ± 1.0	26.2 ± 3.4	37.6 ± 2.6	26.9 ± 4.8
> 9 years’ education	310 (15.7)	20,955 (40.1)	1474 (30.0)	22,739 (38.4)
All births, n (%)	1970 (3.3)	52,304 (88.4)	4916 (8.3)	59,190
Live births, n (%)	1963 (99.6)	52,166 (99.7)	4894 (99.6)	59,023 (99.7)
Males	1020 (51.8)	27,847 (53.2)	2629 (53.5)	31,496 (53.2)
Gestational age (weeks)	39.1 ± 1.7	39.1 ± 1.5	38.8 ± 1.7	39.1 ± 1.5
Birthweight (grams)	3333 ± 507	3448 ± 500	3496 ± 565	3448 ± 507
Preterm birth rate*	94 (4.8)	2006 (3.8)	304 (6.2)	2404 (4.1)
LBW*	87 (4.4)	1435 (2.7)	173 (3.5)	1695 (2.9)
Multi-birth	39 (2.0)	1122 (2.1)	89 (1.8)	1250 (2.1)
Congenital anomalies^a^	13 (0.7)	280 (0.5)	31 (0.6)	324 (0.5)
Cesarean delivery*	911 (46.2)	28,250 (54.0)	3065 (62.3)	32,226 (54.4)
Pregnancy complications*	275 (14.0)	7231 (13.8)	822 (16.7)	8328 (14.1)
HDP*	20 (1.0)	750 (1.4)	120 (2.4)	890 (1.5)
PROM	162 (8.2)	4637 (8.9)	463 (9.4)	0.245
GDM*	14 (0.7)	748 (1.4)	142 (2.9)	904 (1.5)
Anemia*	141 (7.2)	3894 (7.4)	449 (9.1)	4484 (7.6)
Antenatal steroids^f^	27 (30.0)	706 (36.8)	111(37.6)	844 (36.6)
Amniotic fluid volume*				
Normal	1638 (83.1)	44,577 (85.2)	4146 (84.3)	50,361 (85.1)
Polyhydramnios	141 (7.2)	3674 (7.0)	373 (7.6)	4188 (7.1)
Oligohydramnios	191 (9.7)	4054 (7.8)	397 (8.1)	4642 (7.8)
Amniotic contamination				
Normal	1666 (84.6)	45,194 (86.4)	4211 (85.7)	51,071 (86.3)
Grade I	111 (5.6)	2555 (4.9)	258 (5.2)	2924 (4.9)
Grade II	89 (4.5)	1962 (3.8)	171 (3.5)	2222 (3.8)
Grade III	104 (5.3)	2593 (5.0)	276 (5.6)	2973 (5.0)
Apgar score				
1-min ≤7*	76 (3.9)	1320 (2.5)	175 (3.6)	1571 (2.7)
1-min ≤3*	15 (0.8)	284 (0.5)	48 (1.0)	347 (0.6)
5-min ≤7**	25 (1.3)	463 (0.9)	66 (1.3)	554 (0.9)
5-min ≤3**	10 (0.5)	248 (0.5)	35 (0.7)	293 (0.5)
Fetal deaths/stillbirths	7 (0.4)	138 (0.3)	22 (0.4)	167 (0.3)
Deaths at delivery^b^	7 (0.4)	168 (0.3)	23 (0.5)	198 (0.3)
Hospitalized on day 1*	125 (6.3)	2785 (5.3)	354 (7.2)	3265 (5.5)
Early neonatal deaths^c^	4 (0.2)	123 (0.2)	12 (0.2)	139 (0.2)
Late neonatal deaths^d^	0	36 (0.1)	5(0.1)	41 (0.1)
All deaths^e^	11 (0.6)	308 (0.6)	39 (0.8)	358 (0.6)

Values are given in numbers and percentage (% of live births in each maternal age category) or mean ± SD

*Abbreviations*: *LBW* Low birth weight (< 2500 g), *HDP* Hypertensive disorders of pregnancy, *PROM* Premature rupture of membrane, *GDM* Gestational diabetes mellitus

*Percentage of the total number of all live birth (*p* < 0.001)

^a^Including all birth defects and congenital disorders from total births within 7 postnatal days

^b^Including fetal deaths/stillbirth and neonatal deaths immediately at delivery but counted as live births

^c^Including live births died in the first 7 days

^d^Including live births died in the 8 to 28 days after delivery

^e^Including all fetal deaths/stillbirth and all neonatal deaths

^f^Data contain all premature babies received antenatal corticosteroid therapy before delivery

**Table 7:** Maternal education related perinatal status, pregnancy co-morbidities and complications, fetal deaths/stillbirths and neonatal mortality in all births

Education years	< 7	7–9	10–12	>12	Total
Maternal age (years)	28.1 ± 6.1	26.5 ± 5.0	26.8 ± 4.5	27.3 ± 3.9	26.9 ± 4.9
Delayed childbearing*	1094 (16.8)	2348 (7.8)	773 (7.1)	701 (5.9)	4916 (8.3)
All births	6526 (11.0)	29,925 (50.6)	10,941 (18.5)	11,798 (19.9)	59,190
Live births, n (%)	6508 (99.7)	29,855 (99.8)	10,891 (99.5)	11,769 (99.8)	59,023 (99.7)
Males*	3337(51.1)	16,234 (54.2)	5807 (53.1)	6118 (51.9)	31,496 (53.2)
Gestational age (weeks)	39.1 ± 1.5	39.1 ± 1.4	39.0 ± 1.7	39.0 ± 1.6	39.1 ± 1.5
Birthweight (g)	3433 ± 511	3461 ± 498	3426 ± 523	3444 ± 511	3438 ± 507
Preterm birth rate*	236 (3.6)	926 (3.1)	620 (5.7)	622 (5.3)	2404 (4.1)
LBW*	206 (3.2)	711 (2.4)	379 (3.5)	399 (3.4)	1695 (2.9)
Congenital anomalies	37 (0.6)	149 (0.5)	64 (0.6)	74 (0.6)	324 (0.5)
Cesarean delivery	3560 (54.6)	16,745 (56.0)	5936 (54.3)	5985 (50.7)	32,226 (54.4)
Multi-births*	103 (1.6)	488 (1.6)	315 (2.9)	344 (2.9)	1250 (2.1)
Pregnancy complications*	880 (13.5)	3797 (12.7)	1636 (15.0)	2015 (17.1)	8328 (14.1)
Pregnant hypertension*	386 (5.9)	1438 (4.8)	522 (4.8)	574 (4.9)	2920 (4.9)
PROM*	406 (6.2)	2322 (7.8)	1131 (10.3)	1408 (11.9)	5267 (8.9)
GDM*	51 (0.8)	250 (0.8)	235 (2.1)	368 (3.1)	904 (1.5)
Anemia	467 (7.2)	1909 (6.4)	932 (8.5)	1176 (10.0)	4484 (7.6)
Antenatal steroids^f^	106 (47.5)	334 (37.7)	199 (33.3)	205 (34.3)	844 (36.6)
Amniotic fluid volume*					< 0.001
Normal	5688 (87.2)	25,476 (85.1)	9253 (84.6)	9944 (84.3)	50,361 (85.1)
Polyhydramnios	288 (4.4)	2044 (6.8)	856 (7.8)	1000 (8.5)	4188 (7.1)
Oligohydramnios	550 (8.4)	2406 (8.0)	832 (7.6)	854 (7.2)	4642 (7.8)
Amniotic contamination*					< 0.001
Normal	5644 (86.5)	25,697 (85.9)	9477 (86.6)	10,253 (86.9)	51,071 (86.3)
Grade I	424 (6.5)	1695 (5.7)	452 (4.1)	353 (3.0)	2924 (4.9)
Grade II	220 (3.4)	1220 (4.1)	390 (3.6)	392 (3.3)	2222 (3.8)
Grade III	238 (3.6)	1313 (4.4)	622 (5.7)	800 (6.8)	2973 (5.0)
Apgar score					
1-min ≤ 7	155 (2.4)	672 (2.2)	361 (3.3)	383 (3.2)	1571 (2.7)
1-min ≤3	37 (0.6)	149 (0.5)	85 (0.8)	76 (0.6)	347 (0.6)
5-min ≤7**	58 (0.9)	247 (0.8)	137 (1.3)	112 (0.9)	554 (0.9)
5-min ≤3**	29 (0.4)	134 (0.4)	74 (0.7)	56 (0.5)	293 (0.5)
Fetal deaths/stillbirths*	18 (0.3)	70 (0.2)	50 (0.5)	29 (0.2)	167 (0.3)
Deaths at delivery^b^**	19 (0.3)	85 (0.3)	54 (0.5)	40 (0.3)	198 (0.3)
Hospitalized on day 1*	330 (5.1)	1344 (4.5)	745 (6.8)	845 (7.2)	3265 (5.5)
Early neonatal deaths^c^**	10 (0.2)	62 (0.2)	40 (0.4)	27 (0.2)	139 (0.2)
Late neonatal deaths^d^	3	25 (0.1)	5	8 (0.1)	41 (0.1)
All deaths^e^	32 (0.5)	160 (0.5)	101 (0.9)	65 (0.6)	358 (0.6)

Values are given in numbers and percentage (% of all births in each maternal education year category) or mean ± SD

*Abbreviations*: *LBW* Low birth weight (< 2500 g), *HDP* Hypertensive disorders of pregnancy, *PROM* Premature rupture of membrane, *GDM* Gestational diabetes mellitus

* *p* < 0.001; ** *p* < 0.05

^a^Including all birth defects and congenital anomalies from total births within 7 postnatal days

^b^Including fetal deaths/stillbirth and neonatal deaths immediately at delivery but counted as live births

^c^Including live births died in the first 7 days

^d^Including live births died in the 8 to 28 days after delivery

^e^Including all fetal deaths/stillbirth and all neonatal deaths

^f^Data contain all premature babies received antenatal corticosteroid therapy before delivery

### Risk factors for perinatal outcome

Table 8 illustrates the results from uni- (part A) and multivariable (part B) binary logistic regression analysis of risk factors for perinatally associated mortality. By multivariable regression, only BD/CA and Apgar score ≤ 7 at 5 min were the most prominent risk factors whereas multiple births, low GA, amniotic fluid contamination and Apgar score ≤ 7 at 1 min were the modest-to-moderate risk factors for the mortality, all with statistical significance. High risk pregnancy, pregnancy complications and BW were no longer significant risk factors for perinatal mortality compared to the 2010 data analysis [12, 13].

**Table 8:** Uni- and multivariable regression analysis of risk factors for perinatally-associated mortality in all births^a^

A. Univariable logistic regression analysis					
Variables	Category	Mortality (%)	OR	95% CI	*P* values
Hospital level	III	1.1			
	II	0.6	0.503	0.403–0.629	< 0.001
	I	0.2	0.187	0.131–0.266	< 0.001
Sex	Male	0.7			
	Female	0.5	0.777	0.628–0.960	0.019
Multi-births	No	0.5			
	Yes	4.8	9.753	7.347–12.946	< 0.001
Congenital anomalies	No	0.5			
	Yes	13.6	29.303	20.927–41.030	< 0.001
Cesarean delivery	No	0.8			
	Yes	0.4	0.517	0.417–0.640	< 0.001
High risk pregnancy	No	0.2			
	Yes	0.8	4.382	2.939–6.535	< 0.001
Pregnancy complications	No	0.6			
	Yes	0.8	1.383	1.058–1.809	0.018
Low birthweight (< 2500 g)	No	0.3			
	Yes	10.6	37.807	30.543–46.799	< 0.001
Preterm birth (< 37 weeks)	No	0.3			
	Yes	8.5	34.507	27.868–42.728	< 0.001
Maternal education (< 10 years)	No	0.7			
	Yes	0.5	0.720	0.585–0.887	0.002
Maternal age (≥35 years old)	No	0.6			
	Yes	0.8	1.353	0.969–1.888	0.073
AF contamination	No	0.5			
	Yes	1.3	2.741	2.185–3.439	< 0.001
Abnormal amniotic fluid volume	No	0.6			
	Yes	0.8	1.516	1.174–1.958	0.001
First delivery	No	0.5			
	Yes	0.8	1.716	1.392–2.114	<0.001
1-min Apgar (≤7)	No	0.2			
	Yes	16.6	118.149	92.994–150.110	< 0.001
5-min Apgar (≤7)	No	0.2			
	Yes	43.7	391.298	305.437–501.294	< 0.001
B. Multivariable logistic regression analysis					
Variables	Reference^b^	OR	95%CI		*P* values
			Lower	Upper	
Hospital level	I	0.729	0.456	1.166	0.187
	II	0.925	0.670	1.276	0.634
Sex	Male	0.815	0.619	1.074	0.146
Multi-births	No	2.331	1.451	3.744	< 0.001
Congenital anomalies	No	14.699	7.850	27.527	< 0.001
High risk pregnancy	No	1.510	0.941	2.422	0.088
Cesarean delivery	No	0.635	0.480	0.840	0.001
LBW (< 2500 g)	No	1.362	0.875	2.119	0.171
Preterm birth (< 37 weeks)	No	2.606	1.640	4.140	< 0.001
Maternal education (< 10 years)	No	1.005	0.749	1.350	0.972
Maternal age (≥ 35 years old)	No	1.069	0.681	1.678	0.772
AF contamination	No	1.997	1.446	2.758	< 0.001
Abnormal amniotic fluid volume	No	1.322	0.925	1.889	0.125
First delivery	No	0.838	0.616	1.140	0.260
1-min Apgar (≤7)	No	5.922	3.805	9.218	< 0.001
5-min Apgar (≤7)	No	40.593	26.845	61.380	< 0.001

*Abbreviations*: *OR* Odds ratio, *CI* Confidence interval of OR, *AF* Amniotic fluid, *LBW* Low birth weight

^a^Perinatally-associated mortality denotes a ratio of sum of all fetal deaths/stillbirths plus all neonatal deaths versus total births, listed in Tables 1, 2, 3, 4, 5, 6 and 7

^b^As references of the opposite category (level III, female or yes) of corresponding variables in part A

## Discussions

This study mainly illustrated the impact of pregnancy co-morbidities and complications on perinatal and neonatal mortalities (Tables 1 and 2). Compared to the survey in the year of 2010, our 2015 survey revealed important changes in the quality of maternal-fetal and perinatal care paralleled with socio-economic development in emerging regions in China. Above all, the improved perinatal-neonatal outcome is very likely to be associated with highly centralized deliveries at level II and III hospitals [22, 23]. Moreover, pregnancy complications were more timely diagnosed and actively managed in 2015 than in 2010, which probably contributed to improved perinatal outcome despite increasing proportions of pregnancy complications, preterm births and neonatal hospitalization after delivery, as well as an increasing, but still low, rate of antenatal corticosteroid use in preterm births through 2010 to 2015. In 2015 survey, we also noticed a relatively lower proportion of delayed child bearing with dramatically increased proportion of mothers with an education > 9 years. Improved health-care infrastructures in sub-provincial facilities, optimized resource relocation highlighted by centralization of high-risk pregnancies and deliveries, as well as the universal implementation of health insurance to rural families may be the major socioeconomic factors behind the reduced perinatal-neonatal mortality rate in 2015. Our regional birth population based data from both 2010 and 2015 surveys are complementary to nation-wide surveys which mainly included level II-III hospitals [1, 2, 5, 6].

Great regional inequity exists in China, posing a substantial challenge to estimate vital statistics [12, 24]. The previously adopted national surveillance system [1, 2, 4–6] by means of hospital sampling has limitations in representativeness for perinatal care and outcome due to lacking of data from deliveries in most level I to III hospitals. The inclusion of level I (township) hospitals, which had lower rate of preterm births, perinatal and neonatal mortality but consisted around 30% in the total deliveries in the whole region, was the main advantage in contrast to that reported from the nationwide surveillance system for BD/CA and vital statistics in the past decade [1, 2, 4–6]. Notably, neonates born with detectable life signs but died shortly in the delivery room may be misclassified as stillbirths rather than live births in daily obstetric practice as well as in the data presentation from nation-wide surveillance systems. These might account for a much higher stillbirth rate than our data, which should be born in mind not to overestimate the nation-wide situation for stillbirths [14, 25]. As a national birth-population survey including all births in China is very difficult, if possible, to conduct, a representative regional birth-population would be an expedient and plausible alternative for each province to start with. As the sub-provincial regions like Huai’an approaches the national average level in terms of economics represented by GDP, population characteristics and maternal-infant health care standard, we deemed that vital statistics in Huai’an would represent a large proportion of regions in China with similar socioeconomic status, namely emerging regions [12]. With the future establishment of more regional whole birth population registries in various regions, we may be able to more accurately assess the status of perinatal-neonatal healthcare in China.

Compared to the 2010 survey, the incidence of pregnancy complications (14.1%) was significantly enhanced in 2015, mainly contributable to the increase in moderate-to-severe anemia and gestational diabetes mellitus. The incidence of infections during pregnancy in 2010 was 1.8‰ [13], compared to < 2‰ in the current study (data not shown), indicating that infections may not be a leading pregnancy complication in this context. Because deliveries in 2015 were highly centralized in level II and III hospitals (70% of all deliveries), where the accuracy and consistency of diagnosing pregnancy complications can be ensured [15], we speculate that there was inaccuracy in diagnosis and underreporting of pregnancy complications in 2010 [13]. Moreover, the potential perinatal comorbidities and disease burden of neonates born to mothers even without pregnancy complications should not be underestimated. In-depth analyses will be carried out in future studies to explore the severity of pregnancy complications and antenatal treatment in relation to perinatal outcome.

Another problem is the persistently high proportion of cesarean sections lacking clinical indications (Table 5). In particular, 39–50% of VLBW infants with a GA < 32 weeks were born via cesarean delivery (Tables 3 and 4). The high cesarean delivery rate in mothers without pregnancy complications may be partly due to pain in labor and/or disproportionately large fetal body size, especially for the subgroup of infants born full term or post-term with a BW approaching or over 4000 g (Tables 2 and 3) [15]. Whether clinical pathological conditions other than the defined pregnancy complications were implicated in the high rate of non-indicated cesarean section requires further in-depth investigation. For maternal age ≥ 35 years old, the pregnancy complications, preterm birth rate and cesarean deliveries were significantly higher than those < 35 years old, however, risks of low BW, multi-births and BD/CA were modestly high, or not.

Notably maternal mortality rate was reduced from 23/100,000 total births in 2010 to 12/100,000 in 2015 in the study region (Zhu X unpublished data). The main death causes were related to high risk pregnancies, such as amniotic fluid embolism, pulmonary embolism, post-delivery hemorrhage, stroke and sepsis. Most of the maternal deaths occurred in mothers of rural residency, with an education < 9 years and were admitted to level I and II hospitals. With the universal implementation of second-child policy in 2016, we anticipate a rise in the risk of maternal death as well as incidence of pregnancy complications in the following years as a significant proportion of mothers would be at delayed child-bearing age. The incidence of, and perinatal outcome associated with, the pregnancy complications in current survey may serve as a benchmark for future comparison.

The accurate reporting of stillbirths or fetal deaths is a key issue. In both 2010 and 2015 surveys, the perinatal mortality was calculated using the total numbers of stillbirths (and fetal deaths) from 22 weeks of gestation and neonatal death at the first postnatal week as the numerator [12, 13, 15]. In 2010 survey [15], the rates of stillbirths, death immediately after birth and early neonatal deaths were 3.9‰, 1.7‰ and 3.8‰, respectively. By comparison, the corresponding rates in 2015 showed significant reduction, being 2.8‰, 0.5‰ and 2.3‰ respectively (*p* < 0.001). In the US, the incidence of fetal deaths was 6.0‰ in 2013–2014 [26], with infants born in 20–27 weeks of gestation contributing to around half of the fetal deaths. A recent survey of nearly 4 million births in 2012–2014 from 441 sampled hospitals in China revealed a stillbirth rate of 8.8 per 1000 births [14, 25]. However, neither perinatal mortality rate nor death immediately after birth (with resuscitation in the delivery room) was included, indicating serious limitations in the methodology and may produce biased or misleading results in the causes of perinatal-neonatal deaths [1, 2, 5, 6]. Based on our studies, we estimate a nation-wide stillbirth rate to be around 5–7‰ when neonates with a GA ≥ 25 weeks were taken into account. Controversies remain as whether the definition of stillbirths should start from the gestation of 22 or 25 weeks instead of 28 weeks, and the diagnosis of death immediately after birth in live births may be difficult in level I-III facilities [15]. The definition of perinatal mortality used in our studies is not yet, but preferably, adopted by the official reporting system for birth related vital statistics from the Chinese national health authority [17]. The US vital statistics [26] in 2014 demonstrated 3,988,076 total births by complete birth registry, compared with 16–17 millions in China in 2014–2017 by estimation from the sampling data [17], corresponding to a birth rate around 12.5‰ in the US vs. 11.8‰ in China [16]. The preterm birth rate in the US declined from 12% in 2000–2010 to < 10% in 2013–2014, whereas in Europe and other industrialized countries, the preterm birth rates were reported to be 6–8% [26]. Other developing country in Asia, such as Vietnam, has shown a similar preterm birth rate (< 5%) in emerging regions [27] as in our study. This corroborates the roles of both biological and socioeconomic factors in vital statistics and perinatal care.

As consistent to the 2010 survey, maternal age and education years as socioeconomic factors continued to impact on the regional perinatal and neonatal outcome [28–31]. Our data revealed that the pregnancies with age < 20 years old had modestly increased rates in low birth weight, preterm births, and CA/BD, compared to the normal age (20–34 years old) pregnancies (Table 6). In the uni- and multivariable logistic regression analysis, the pregnancy with delayed child bearing (age ≥ 35 years old) tended not to significantly contribute to the worse perinatal outcomes such as fetal death/stillbirths and neonatal deaths (Table 8). Likewise, the dramatic improvement in the maternal education years in 2015 survey should have also indirectly contributed to the reduction of worse perinatal outcome (Tables 7 and 8). Taking together the results from uni- and multivariable logistic regression analysis, the impact of perinatal risks on the changes in perinatal and neonatal outcome was associated with maternal biological, clinical and socioeconomic status over the two epochs, reflecting the improved management of pregnancy complications and other major perinatal risks as well as infrastructure of the perinatal network, as evidenced by both surveys.

In regard to limitations in the study design, the causal relationship between pregnancy complications and perinatal outcomes could not be elicited. However, it provides comprehensive information revealing the potential impact of pregnancy complications on perinatal outcomes at the birth population level. Given a serious lack of data in maternal-fetal and neonatal care in China, this study may be valuable to address this gap of knowledge. Moreover, severity of pregnancy complications was not stratified, and detailed data concerning service quality associated with management of high-risk pregnancies and pregnancy complications in the included hospitals was insufficient too, which limits in-depth investigation such as cost-effectiveness assessment for the relationship or efficiency of health care policies. As Huai’an is considered to be representative of the emerging regions, information on the medical infrastructure and the standard of care in the region may help to better evaluate the current status in China. It is desirable that birth populations of sub-provincial regions in each province be first investigated, thereafter extending to the whole province level. Gradually a comprehensive national perspective might be achieved.

## Conclusion

In conclusion, results from this survey revealed the relationship between high risk pregnancies and perinatal outcome, and strengthened the reliability and feasibility of the regional birth-population based study concept and protocol. The regional perinatal information system was tightly and efficiently implemented as the mainstay of the two epochs, especially in the current survey. It may provide valuable information for improvement in maternal-fetal and neonatal care in China.

## Additional file

Additional file 1: **Table S1.** List of member hospitals (level II and III) of the Huai’an Perinatal-Neonatal Study group. (DOCX 15 kb)

## Abbreviations

BD: Birth defects
BW: Birth weight
CA: Congenital anomalies
GA: Gestational age
GDP: Gross domestic production per capita
HDP: Hypertensive disorders of pregnancy
