# Supplementary material for: High risk pregnancy associated perinatal morbidity and mortality: a second birth population-based survey in Huai’an in 2015
Source: BMC Pregnancy Childbirth. 2019 Jul 3;19:224. doi: 10.1186/s12884-019-2323-6 (PMC6609375; doi:10.1186/s12884-019-2323-6)
Supplement: Supplementary file 1 — Table S1. List of member hospitals (level II and III) of the Huai’an Perinatal-Neonatal Study group. (DOCX 15 kb) [file 12884_2019_2323_MOESM1_ESM.docx]

List of member hospitals of Huai’an Perinatal Network Study Group

Level III:

Huai’an Women and Children's Hospital

Huai’an First People's (General) Hospital

Huai’an Second People's (General) Hospital

Huai’an Traditional Chinese Medicine Hospital

PLA 82nd General Hospital (uniformed service)

Huai’an District Hospital

Level II:

Huai’an District Maternity Hospital

Huai’an District Xinqu Hospital

Huai’an City Xiehe Hospital

The First Division of Huaian First People's (General) Hospital

Huaiyin District Hospital

Huaiyin District Maternity Hospital

Lianshui County People's (General) Hospital

Lianshui Second People's (General) Hospital

Lianshui County Third People's (General) Hospital

Lianshui County Traditional Chinese Medicine Hospital

Hongze County Traditional Chinese Medicine Hospital

Hongze County Maternity Hospital

Xuyi County People's (General) Hospital

Xuyi County Traditional Chinese Medicine Hospital

Jinhu County People's (General) Hospital

Jinhu County Traditional Chinese Medicine Hospital
